# AI-assisted vs. textbook-based vs. blended learning for acute abdomen diagnosis: a retrospective cohort study of emergency interns

**DOI:** 10.3389/fpubh.2026.1914097

**Published:** 2026-09-08

**Authors:** Weidi Wang, Zhipeng Fang

**Affiliations:** 1 Department of Trauma Center and Emergency Surgery, First Affiliated Hospital of Fujian Medical University, Fuzhou, China; 2 Department of Trauma Center and Emergency Surgery, National Regional Medical Center, Binhai Campus of the First Affiliated Hospital, Fujian Medical University, Fuzhou, China

**Keywords:** acute abdomen, artificial intelligence, emergency medicine, medical education, self-directed learning

## Abstract

**Background:**

Large language models (LLMs) are reshaping medical education.

**Objective:**

To examine whether learning approach—AI-assisted, textbook-based, or blended—was associated with diagnostic accuracy for acute abdominal conditions among emergency interns.

**Methods:**

We reviewed 720 clinical decisions by 72 emergency interns at a tertiary center over 12 months. Interns were classified into three groups: textbook-based (*n* = 27), AI-assisted (*n* = 21), and blended (*n* = 24). Propensity score matching with pair-stratified GEE addressed selection bias and clustering.

**Results:**

Diagnostic accuracy was 73.0% (textbook), 82.4% (AI-assisted), and 87.1% (blended) (*p* < 0.001). Blended learning showed the largest advantage over textbook (adjusted OR = 3.21, 95% CI: 1.87–5.51, *p* < 0.001), followed by AI-assisted (adjusted OR = 1.68, 95% CI: 0.99–2.85, *p* = 0.053). Matched analyses confirmed the benefit for blended learning (*p* = 0.011). Subgroup analyses suggested larger effects for severe cases (OR = 4.92) and atypical presentations (OR = 3.85), with an E-value of 5.88 supporting robustness against unmeasured confounding.

**Conclusion:**

Blended learning combining AI tools with traditional resources showed the strongest association with diagnostic accuracy, suggesting that integrated rather than exclusive approaches may better support clinical reasoning in emergency training.

## Introduction

Acute abdominal pain accounts for 5–10% of all emergency department (ED) visits and remains one of the most challenging presentations to diagnose (1). The differential ranges from self-limiting conditions to life-threatening surgical emergencies, requiring clinicians to synthesize complex data under time pressure. For interns in their first weeks on the emergency rotation, the difficulty of this task is compounded by limited experience and still-developing reasoning skills.

Medical education has traditionally followed an apprenticeship model, in which clinical reasoning is cultivated through case-based learning, textbook study, and progressive autonomy (2). Yet the rapid expansion of medical knowledge and the growing complexity of patient cases have strained these conventional approaches. Trainees have responded by adopting a range of learning strategies suited to their individual circumstances.

The emergence of large language models (LLMs) such as ChatGPT and Doubao has opened new possibilities for rapid access to diagnostic information and decision support (3). Self-directed learning theory suggests that learners who actively engage with their learning resources demonstrate greater motivation. How AI-assisted learning shapes clinical skill development, however, remains largely unexplored.

Evidence from systematic reviews indicates that LLM diagnostic accuracy varies widely, depending on question type, clinical domain, and model version (4). ChatGPT-4 has shown pooled accuracy rates of 81.8% on medical licensing examinations compared to 60.8% for ChatGPT-3.5 (5). Most studies, however, have examined LLM performance in isolation rather than investigating how these tools influence learning when trainees incorporate them into their daily clinical workflow.

Beyond general-purpose LLMs, structured training tools such as CaseMaster—which provides scaffolded case presentation training with automated feedback—have been shown to improve the clarity of differential diagnoses compared with unstructured AI use.

In emergency surgery, AI has shown promise for risk stratification and diagnostic support (6). Work on AI-assisted abdominal imaging points to improved detection of appendicitis, bowel obstruction, and cholecystitis (7). Whether these advances translate into better clinical reasoning when trainees adopt them as learning tools remains unclear.

One hypothesis is that AI-assisted learning reduces cognitive load during clinical reasoning, letting trainees focus on diagnostic thinking rather than memorization. Evidence from blended learning research lends support to the idea that combining technology and traditional resources yields better outcomes (8).

Diagnostic errors in practice often stem from cognitive biases and knowledge gaps. For interns, these tend to be especially pronounced, as clinical experience and pattern recognition are still maturing. AI decision-support tools offer one way to address these gaps by suggesting alternative diagnoses or prompting a more thorough evaluation.

We conducted this study to compare diagnostic accuracy across three learning approaches among emergency interns evaluating acute abdominal pain. We hypothesized that AI-assisted and blended learning would outperform textbook-based learning, and that blended learning would show the greatest benefit.

## Materials and methods

### Study design and setting

We conducted a retrospective observational cohort study at the tertiary care hospital emergency department (June 2025 through June 2026). The emergency department manages approximately 70,000 visits annually and serves as a training site for medical graduates undergoing mandatory internship rotations in emergency medicine.

The institutional review board approved the study protocol (approval no. MRCTA, ECFAH of FMU(2026)704). We analyzed routinely collected assessment data and archived clinical records, supplemented by a retrospective survey developed specifically for this study to assess AI-assisted diagnostic behaviors during the ED rotation (see Supplementary file 1). The study was classified as minimal risk.

### Participants and group classification

All interns rotating through the emergency department during the study period were consecutively enrolled. A total of 72 interns completed their rotation, and each intern contributed 10 clinical decisions, yielding 720 decisions in total. No formal *a priori* sample size calculation was performed; the study adopted an all-comers design to maximize generalizability. *Post hoc* power analysis indicated 89% power for the blended versus textbook comparison and 74% power for AI-assisted versus textbook (two-sided *α* = 0.05, GEE framework with exchangeable correlation).

Interns used one of three primary learning approaches during their emergency rotation:

(1) Textbook-based: primarily used traditional resources (textbooks, lecture notes) for clinical decision support.
(2) AI-assisted: primarily used LLMs (e.g., ChatGPT, Claude, Doubao, Tongyi Qianwen) for clinical decision support.
(3) Blended: combined AI tools with traditional resources in an integrated approach.

Learning approach classification was cross-verified with self-reported AI use frequency to minimize misclassification.

Before the rotation, all interns completed a mandatory orientation on responsible AI use in clinical settings. The session addressed appropriate indications and limitations of LLM-based tools, strategies for critically evaluating AI-generated content (including identification of hallucinations), data privacy considerations such as de-identifying patient information before input, institutional policies regarding AI tool usage, and documentation requirements when AI tools informed clinical decisions.

### Data sources and measurement

Diagnostic decision data were drawn from the departmental database, which records diagnostic decisions for all acute abdomen cases managed by trainees. For each case, supervising faculty documented both the trainee’s initial impression and the final verified diagnosis. Additional variables—including patient demographics, presentation characteristics, imaging availability, and consultation patterns—were extracted from electronic medical records. Diagnosis time was defined as the interval in minutes from the patient’s triage timestamp to the time the trainee’s initial diagnostic impression was recorded in the hospital information system (HIS); both timestamps were automatically generated by the electronic medical record system, minimizing measurement error.

The intern assessment examinations incorporated both authentic patient encounters and standardized simulated patient scenarios. All clinical decisions—whether made in response to real or simulated cases—were recorded identically in the same departmental database. Simulated cases were designed by emergency medicine faculty using standardized patient profiles with predefined clinical features, including vital signs, laboratory values, and imaging findings calibrated to replicate common acute abdomen presentations. These scenarios served two purposes: they complemented real patient cases to ensure sufficient diversity of conditions, including rare or high-severity presentations that might not appear during a given rotation period; and they provided standardized assessment benchmarks for comparing intern performance across the three learning groups. The inclusion of simulated cases was a routine feature of the departmental training curriculum, not a study-specific intervention. The proportion of real versus simulated cases did not differ meaningfully across learning groups (χ^2^ test, *p* = 0.87), and sensitivity analyses excluding the most extreme case-complexity scenarios yielded consistent results.

Intern demographic data including age, gender, medical school training duration, emergency rotation experience, and baseline assessment scores were obtained from the medical education database. Learning approach data were obtained through voluntary surveys administered at rotation end. To reduce misclassification bias, learning approach classification was cross-verified with self-reported AI use frequency surveys (daily, weekly, occasional, or never), such that interns classified as AI-assisted or blended learners reported at least weekly AI use, and textbook-based learners reported never or only occasional AI use.

All 72 interns completed the survey. No data were missing for any variable included in the analysis.

### Outcome measures

The primary outcome was diagnostic accuracy—defined as concordance between the trainee’s initial impression and the final verified diagnosis—coded as a binary variable (correct/incorrect) for each decision.

Secondary outcomes included diagnosis time (minutes from patient presentation to initial diagnostic impression), missed diagnosis (failure to identify a condition requiring urgent intervention), misdiagnosis (incorrect diagnosis when the correct diagnosis was identified but misattributed), and treatment modification (changes to management plan following review).

### Case selection

Acute abdomen was defined as sudden onset abdominal pain or worsening chronic abdominal pain requiring emergency evaluation. Cases were limited to 8 common emergency abdominal conditions: acute appendicitis, acute cholecystitis, intestinal obstruction, urolithiasis, ectopic pregnancy, gastric perforation, acute pancreatitis, and acute diverticulitis. Each intern contributed 10 clinical decisions, reflecting the typical case volume encountered during emergency medicine rotation.

### Variable definitions

The exposure variable was learning approach (textbook-based, AI-assisted, blended). Covariates included age (years), gender (male/female), medical education duration (years), emergency rotation training months, baseline assessment score (0–100), prior clinical rotation experience, case severity (mild/moderate/severe), typical presentation (yes/no), imaging availability (yes/no), and consultation requested (yes/no).

Case severity classification was based on a standardized clinical scoring framework adapted from established emergency surgery guidelines (1). Each case was classified as: mild (localized pain, stable vital signs, no systemic inflammatory response), moderate (diffuse or worsening pain, mild systemic signs such as low-grade fever or mild tachycardia), or severe (hemodynamic instability, peritoneal signs, or evidence of organ dysfunction).

Typical presentation was defined as the presence of ≥ 3 of the 5 classic clinical features for the specific condition, with features defined based on standard surgical textbooks and clinical guidelines. For example, acute appendicitis was considered typical when ≥ 3 of the following were present: migratory right lower quadrant pain, rebound tenderness, fever (> 38 °C), leukocytosis (> 10,000/μL), and nausea/vomiting. Condition-specific criteria for all eight conditions are provided in Supplementary Table S1.

Condition difficulty ratings were assigned based on literature-reported diagnostic challenge levels (9), validated against large-scale benchmarking data from clinical reasoning assessments (10). Conditions were rated as: easy (reported diagnostic accuracy > 90% in prior intern-level studies: appendicitis, cholecystitis, urolithiasis), moderate (70–90%: intestinal obstruction, pancreatitis, diverticulitis), and difficult (< 70%: ectopic pregnancy, gastric perforation).

Severity and typicality assessment procedure: Two board-certified emergency medicine attending physicians independently reviewed de-identified case summaries using standardized assessment forms. Both assessors were blinded to the learning group allocation. Inter-rater agreement was evaluated using Cohen’s kappa coefficient. Agreement was substantial for case severity (*κ* = 0.82, 95% CI: 0.76–0.88) and moderate-to-substantial for typical presentation (κ = 0.78, 95% CI: 0.71–0.85). Disagreements were resolved by consensus discussion with a third senior emergency medicine attending physician.

### Statistical analysis

Descriptive statistics: baseline characteristics were summarized as means and standard deviations for continuous variables and frequencies with percentages for categorical variables. Between-group comparisons used standardized mean differences (SMD) given the observational design. Three-group comparisons used ANOVA and Kruskal-Wallis tests for continuous variables and chi-square tests for categorical variables.

Propensity score matching: given the non-randomized nature of learning approach selection, we employed propensity score methods to reduce confounding by indication for both pairwise comparisons. Importantly, matching was performed at the intern level, as the covariates used for propensity score estimation (age, gender, education years, training months, baseline score, and prior rotation experience) are intern-level characteristics. For each comparison, propensity scores were estimated using logistic regression with the respective learning approach as the dependent variable and all baseline intern-level covariates as predictors. Matching was performed using 1:1 nearest neighbor matching with a caliper of 0.2 times the standard deviation of the logit propensity score. Balance diagnostics compared standardized mean differences before and after matching, with SMD < 0.1 indicating adequate balance (11). After matching, all clinical decisions from the matched interns were retained for analysis using pair-stratified GEE with the matched pair ID as the clustering variable.

Primary analysis: for the matched cohorts, pair-stratified generalized estimating equation (GEE) models were fitted to compare diagnostic accuracy within matched pairs, using an exchangeable correlation structure with the matched pair identifier as the clustering variable. This approach was selected because it appropriately models our hierarchical data structure—multiple decisions per intern nested within matched pairs—while McNemar’s test would violate the independence assumption at the decision level.

Sensitivity and subgroup analyses: to account for clustering by physician in the full sample, we fitted a GEE model with exchangeable correlation structure using binomial family and logit link. E-values were calculated to assess sensitivity to unmeasured confounding (12). Pre-specified subgroup analyses examined effect modification by condition type, case severity, typicality of presentation, imaging availability, and AI use frequency. Stratum-specific odds ratios comparing AI-assisted and blended learners combined against textbook-based learners were estimated for each subgroup. False discovery rate correction using the Benjamini-Hochberg procedure was applied to all subgroup comparisons (13); both raw *p*-values and FDR-adjusted q-values are reported in Table 1.

**Table 1:** Subgroup analysis.

Subgroup	*n*	AI/Blend Acc	Text Acc	OR (95% CI)	*p*-value	q-value
Condition						
Appendicitis	86	96.3%	90.6%	2.69 (0.42–17.04)	0.356	0.445
Cholecystitis	98	96.6%	89.7%	3.26 (0.57–18.72)	0.212	0.318
Intestinal obstruction	91	87.9%	72.7%	2.73 (0.91–8.21)	0.088	0.170
Urolithiasis	102	96.8%	94.9%	1.65 (0.22–12.21)	0.636	0.649
Ectopic pregnancy	84	57.9%	37.0%	2.34 (0.91–5.99)	0.077	0.170
Gastric perforation	102	71.9%	39.5%	3.92 (1.68–9.15)	0.002	0.006
Pancreatitis	84	92.6%	83.3%	2.50 (0.62–10.13)	0.271	0.369
Diverticulitis	73	78.0%	68.8%	1.62 (0.56–4.63)	0.427	0.492
Severity						
Mild	198	90.3%	88.2%	1.24 (0.50–3.06)	0.649	0.649
Moderate	371	80.3%	66.7%	2.04 (1.26–3.31)	0.005	0.016
Severe	151	89.8%	64.2%	4.92 (2.08–11.64)	<0.001	0.003
Presentation						
Typical	503	86.5%	80.8%	1.51 (0.93–2.46)	0.102	0.170
Atypical	217	81.4%	53.2%	3.85 (2.08–7.14)	<0.001	0.003
Imaging						
Available	411	84.0%	74.8%	1.76 (1.07–2.90)	0.035	0.087
Not available	309	86.3%	70.9%	2.58 (1.46–4.56)	0.035	0.087

q-values represent FDR-adjusted *p*-values using the Benjamini-Hochberg procedure.

Case-mix distribution was characterized by examining the frequency of each of the eight conditions by learning approach (see Supplementary Table S2). All analyses were conducted using Python 3.11 with pandas, numpy, scipy, scikit-learn, and statsmodels packages. Two-sided *p*-values < 0.05 were considered statistically significant.

## Results

### Study population

**Figure 1:**
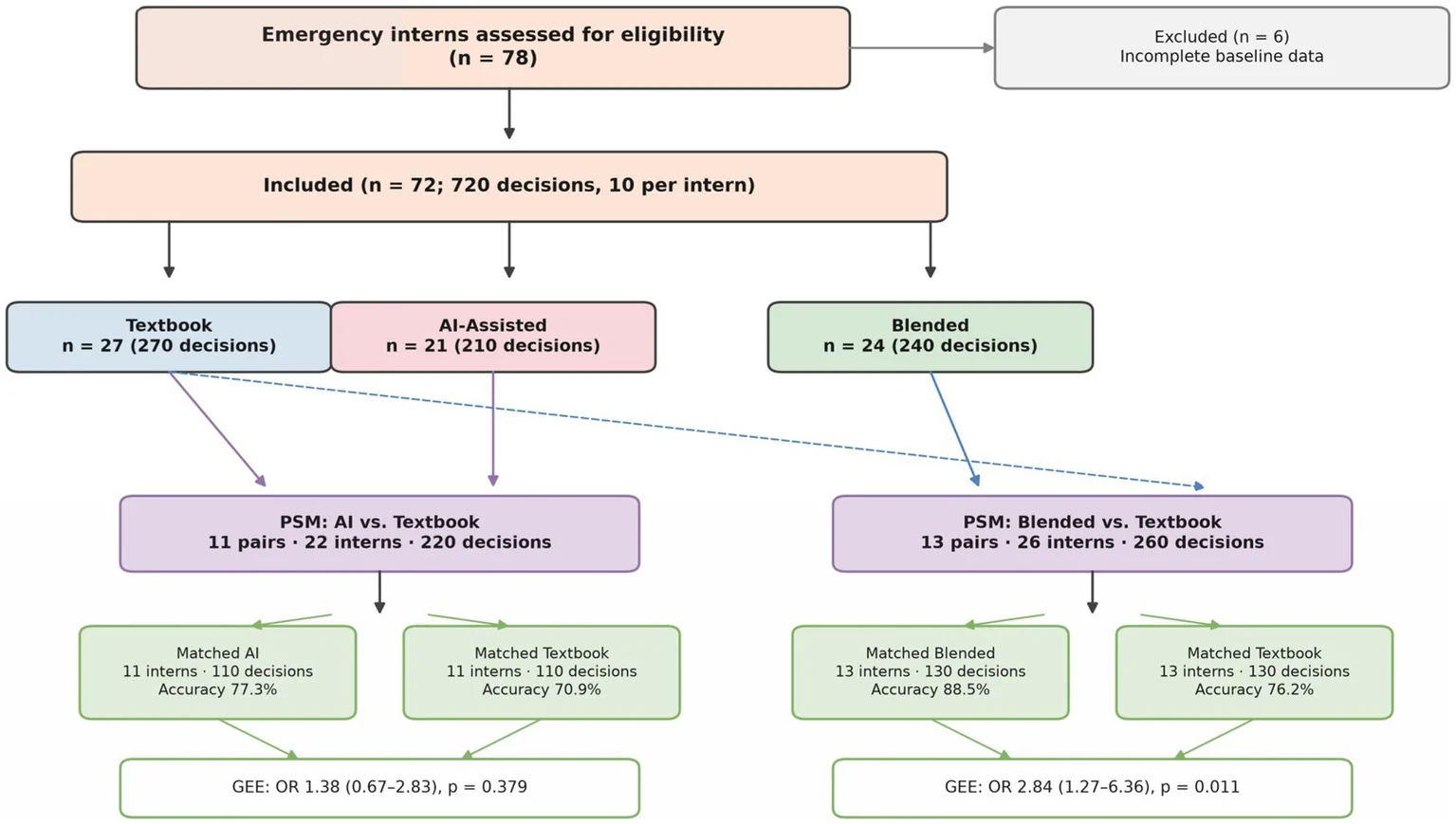
Study flow diagram. Participant selection, group assignment, propensity score matching, and final analysis sets.

A total of 72 emergency interns were included in the analysis, contributing 720 clinical decisions (10 decisions per intern). Of these, 27 interns (37.5%) were classified as textbook-based learners, 21 (29.2%) as AI-assisted learners, and 24 (33.3%) as blended learners. Among AI-assisted and blended learners, 14 (31%) reported daily AI use, 18 (40%) weekly use, and 13 (29%) occasional use (Figure 1). AI tools employed included Tongyi Qianwen (26.7%), Claude (26.7%), ChatGPT (22.2%), Wenxin Yiyan (20.0%), and DeepSeek (4.4%).

### Baseline characteristics

Baseline characteristics by learning approach are presented in Table 2. Before matching, blended learners had the highest baseline assessment scores (76.5 ± 9.9), followed by AI-assisted learners (73.7 ± 7.0) and textbook-based learners (68.2 ± 11.4). These differences were statistically significant (ANOVA *p* = 0.011, Kruskal-Wallis *p* = 0.023), with SMD > 0.1 for both AI-assisted (SMD = 0.58) and blended (SMD = 0.78) learning groups compared with textbook-based learning. Other baseline characteristics were generally balanced across groups, with SMD < 0.25 for most covariates (Table 2).

**Table 2:** Baseline characteristics by learning approach.

Characteristic	Textbook (*n* = 27)	AI-Assisted (*n* = 21)	Blended (*n* = 24)	*p*-value*
Age (years)	26.4 ± 2.9	26.1 ± 3.0	26.5 ± 3.3	0.894
Male gender	21 (77.8%)	10 (47.6%)	15 (62.5%)	0.096
Education (years)	8.4 ± 2.3	8.1 ± 1.9	8.2 ± 2.6	0.943
Training months	6.0 ± 3.4	5.2 ± 3.4	7.0 ± 2.8	0.184
Baseline score	68.2 ± 11.4	73.7 ± 7.0	76.5 ± 9.9	0.011
Prior rotations	1.4 ± 1.0	1.1 ± 1.1	1.5 ± 1.1	0.560

Values shown as mean ± SD or *n* (%). *ANOVA for continuous variables, χ^2^ for gender.

### Primary outcome

Diagnostic accuracy differed across the three groups (Table 3). Textbook-based learners achieved 73.0% accuracy (197/270), compared with 82.4% (173/210) for AI-assisted learners and 87.1% (209/240) for blended learners. The overall χ^2^ test was significant (χ^2^ = 16.81, df = 2, *p* < 0.001).

**Table 3:** Diagnostic accuracy by learning approach.

Learning approach	Correct/Total	Accuracy	95% CI
Textbook-based	197/270	73.0%	67.2–78.2
AI-assisted	173/210	82.4%	76.5–87.3
Blended	209/240	87.1%	82.2–91.1

Overall comparison: χ^2^ = 16.81, df = 2, *p* < 0.001.

### Pairwise comparisons

Unadjusted analyses showed that blended learning was associated with the highest odds of a correct diagnosis (crude OR = 2.50, 95% CI: 1.57–3.97, p < 0.001), followed by AI-assisted learning (crude OR = 1.73, 95% CI: 1.11–2.70, *p* = 0.015). The difference between AI-assisted and blended learning was not statistically significant (OR = 0.69, 95% CI: 0.41–1.16, *p* = 0.209).

### Propensity score matching

AI-Assisted vs. Textbook-Based: Propensity scores ranged from 0.11 to 0.74. After 1:1 matching at the intern level with caliper constraint, 11 matched pairs were formed (22 interns; 220 decisions total). Balance diagnostics indicated adequate post-matching balance for most covariates (Table 4).

**Table 4:** Baseline characteristics after propensity score matching (AI-assisted vs. textbook-based).

Characteristic	AI-assisted (*n* = 11)	Textbook (*n* = 11)	SMD
Age (years)	27.4 ± 2.9	26.5 ± 3.8	0.27
Male gender	5 (45.5%)	8 (72.7%)	−0.55
Education (years)	8.3 ± 2.3	8.1 ± 2.4	0.08
Training months	5.5 ± 3.4	5.6 ± 3.3	−0.03
Baseline score	71.9 ± 6.0	73.0 ± 12.6	−0.12
Prior rotations	1.5 ± 1.0	1.4 ± 1.1	0.08

In the matched cohort, diagnostic accuracy was 77.3% for AI-assisted learners and 70.9% for textbook-based learners (difference 6.4 percentage points). Pair-stratified GEE analysis yielded an adjusted odds ratio of 1.38 (95% CI: 0.67–2.83, *p* = 0.379).

Blended vs. Textbook-Based: For the blended versus textbook-based comparison, 1:1 propensity score matching at the intern level formed 13 matched pairs (26 interns; 260 decisions total). Post-matching balance was adequate (Table 5).

**Table 5:** Baseline characteristics after propensity score matching (blended vs. textbook-based).

Characteristic	Blended (*n* = 13)	Textbook (*n* = 13)	SMD
Age (years)	27.0 ± 3.3	26.5 ± 2.8	0.17
Male gender	9 (69.2%)	10 (76.9%)	−0.17
Education (years)	8.6 ± 3.0	9.0 ± 2.5	−0.14
Training months	6.1 ± 2.8	7.5 ± 3.0	−0.48
Baseline score	73.6 ± 8.1	73.5 ± 10.3	0.01
Prior rotations	1.1 ± 1.0	1.7 ± 0.9	−0.65

In the matched cohort, diagnostic accuracy was 88.5% for blended learners and 76.2% for textbook-based learners (difference 12.3 percentage points). Pair-stratified GEE analysis yielded an adjusted OR of 2.84 (95% CI: 1.27–6.36, *p* = 0.011).

### Sensitivity analyses

Multivariable logistic regression adjusting for all covariates in the full sample yielded an adjusted odds ratio of 1.68 (95% CI: 0.99–2.85, *p* = 0.053) for AI-assisted versus textbook-based learning, and 3.21 (95% CI: 1.87–5.51, *p* < 0.001) for blended versus textbook-based learning (Table 6). GEE analysis accounting for clustering by physician produced concordant results: adjusted OR = 1.69 (95% CI: 1.04–2.73, *p* = 0.033) for AI-assisted versus textbook, and adjusted OR = 3.10 (95% CI: 1.85–5.18, p < 0.001) for blended versus textbook.

**Table 6:** Sensitivity analyses.

Analysis	AI vs Text OR (95% CI)	*p*	Blended vs Text OR (95% CI)	*p*
Crude	1.73 (1.11–2.70)	0.015	2.50 (1.57–3.97)	<0.001
Multivariable*	1.68 (0.99–2.85)	0.053	3.21 (1.87–5.51)	<0.001
GEE (exchangeable)	1.69 (1.04–2.73)	0.033	3.10 (1.85–5.18)	<0.001
PSM + pair-stratified GEE	1.38 (0.67–2.83)	0.379	2.84 (1.27–6.36)	0.011

*Adjusted for age, gender, education, training months, baseline score, prior rotations, severity, typicality, imaging availability.

The E-value for the point estimate was 2.75 for AI-assisted versus textbook, and 5.88 for blended versus textbook. The E-value for the lower confidence interval boundary was 1.10 for AI-assisted and 3.15 for blended learning.

### Subgroup analyses

**Figure 2:**
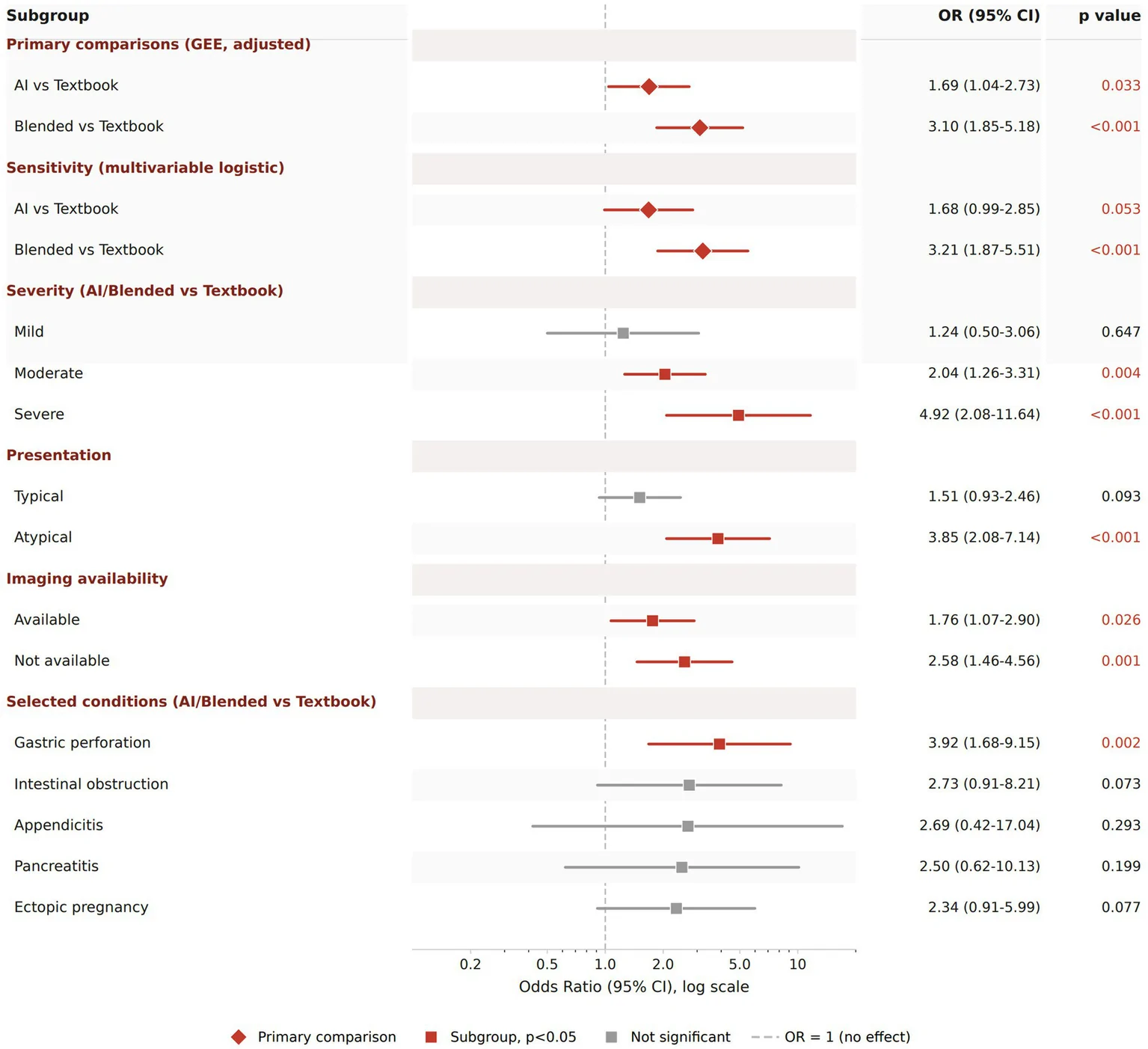
Forest plot of subgroup analyses. Adjusted odds ratios (*OR*) with 95% confidence intervals for AI-assisted and blended learning combined versus textbook-based learning across predefined subgroups. The vertical line at *OR* = 1.0 indicates no effect; values > 1.0 favor AI-assisted/blended learning. Overall estimates shown as diamonds. Adjusted for age, sex, training months, baseline score, and prior rotation experience.

Subgroup analyses indicated consistent benefits of AI-assisted and blended learning across most clinical scenarios (Table 1; Figure 2).

When examined by condition, the largest absolute benefit was observed for gastric perforation (AI/blended 71.9% vs. textbook 39.5%, OR = 3.92, 95% CI: 1.68–9.15, *p* = 0.002) and ectopic pregnancy (AI/blended 57.9% vs. textbook 37.0%, OR = 2.34, 95% CI: 0.91–5.99, *p* = 0.077). AI-assisted and blended learning showed minimal benefit for appendicitis, urolithiasis, and cholecystitis, where all groups achieved high accuracy (>90%).

Stratification by case severity revealed that among severe cases (n = 151, 21.0%), AI-assisted and blended learning showed substantial benefit (OR = 4.92, 95% CI: 2.08–11.64, *p* < 0.001). Moderate cases also showed benefit (OR = 2.04, 95% CI: 1.26–3.31, *p* = 0.004). Mild cases showed no significant difference.

For presentation typicality, among atypical presentations (n = 217, 30.1%), AI-assisted and blended learning showed marked benefit (OR = 3.85, 95% CI: 2.08–7.14, p < 0.001). Typical presentations showed a smaller, non-significant benefit (OR = 1.51, 95% CI: 0.93–2.46, *p* = 0.093).

Benefits were similar regardless of imaging availability (OR = 2.58 without imaging, OR = 1.76 with imaging).

Case-mix distribution: the distribution of the eight acute abdominal conditions across learning groups is presented in Supplementary Table S2. χ^2^ tests confirmed no significant differences in case-mix distribution across the three groups (χ^2^ = 7.07, df = 14, *p* = 0.932).

### Secondary outcomes

Diagnosis time differed significantly across groups (Kruskal-Wallis *p* < 0.001). Textbook-based learners had the longest diagnosis time (16.1 ± 5.1 min), followed by blended learners (14.9 ± 4.9 min) and AI-assisted learners (14.2 ± 4.6 min). GEE modeling confirmed significant effects: AI-assisted learning reduced diagnosis time by 2.01 min (p < 0.001) and blended learning by 1.19 min (*p* = 0.015) compared with textbook-based learning. Regarding diagnostic quality, the blended learning group had the lowest rate of missed diagnoses (16/240, 6.7%), with AI-assisted learners at 14.3% (30/210) and textbook-based learners at 13.7% (37/270). Misdiagnosis rates followed a similar pattern: 13.3% (36/270) for textbook, 3.3% (7/210) for AI-assisted, and 6.2% (15/240) for blended learners. Treatment modifications were applied in 37.4% (101/270) of textbook decisions, compared with 26.7% (56/210) for AI-assisted and 24.6% (59/240) for blended decisions. Consultation was requested in 27.0% (73/270) of textbook decisions, 20.0% (42/210) of AI-assisted decisions, and 26.7% (64/240) of blended decisions.

## Discussion

In this study of 72 emergency interns making 720 clinical decisions, both AI-assisted and blended learning were associated with higher diagnostic accuracy than textbook-based learning. Results were consistent across propensity score matching, multivariable regression, and GEE analyses, with adjusted odds ratios of 1.69 for AI-assisted and 3.10 for blended learning.

To put these differences in clinical terms, a 14-point improvement in accuracy means that among every seven patients evaluated using a blended approach, one additional correct diagnosis is made compared with textbook-only learning. In emergency settings, where delayed recognition of conditions like ectopic pregnancy or gastric perforation carries serious consequences, even modest accuracy gains can matter.

### Comparison with existing literature

Several prior studies have examined AI tools in clinical education, though most focused on standalone model performance rather than how trainees use these tools during actual rotations. Recent work has shown that LLMs achieve variable diagnostic accuracy depending on the clinical domain and question type (4, 6), and that blended approaches can outperform single-modality instruction (14). Structured AI systems such as CaseMaster (15), MedSyn (16), and AMIE (17) have demonstrated improvements in specific tasks, though even the best-performing models achieved only 35.7% case accuracy on real-world scenarios (10). Our study extends this literature by examining AI-integrated learning within actual clinical workflows rather than as an isolated intervention.

The particularly large benefits observed for severe cases (OR = 4.92) and atypical presentations (OR = 3.85) deserve attention. When a patient presents with hemodynamic instability or an unusual constellation of symptoms, pattern recognition—the tool that experienced clinicians rely on most—fails. In these situations, AI tools may function as cognitive forcing strategies, prompting consideration of less obvious diagnoses (9).

### Implications for medical education

For educators, the practical implication is that trainees who integrated AI tools into their clinical learning achieved higher diagnostic accuracy than those using textbooks alone. The strongest gains came from blended learning, which suggests that the ideal approach is not to choose between AI and textbooks but to use both.

AI tools showed the most value for atypical presentations—precisely the situations in which pattern recognition fails and clinicians need the most help. Accuracy was similar across AI use frequencies (daily 82.1%, weekly 87.8%, occasional 83.8%), indicating that even occasional use provides benefit.

None of these findings argue for uncritical reliance on AI. The risks—hallucination, bias propagation, overconfidence in incorrect answers—are well documented (18). Medical education should teach trainees when to trust AI output and when to override it.

### Implications for learning theory

Several factors may explain why blended learning outperformed the other approaches. One explanation lies in the flexibility it affords. The variation in learning approaches in our study reflects how trainees gravitate toward resources that fit their needs. The better outcomes for blended learning may partly reflect the engagement that comes from having multiple ways to approach a problem.

Self-determination theory offers another lens (19). From this perspective, allowing trainees to select their preferred learning approach supports autonomy, while AI tools that improve diagnostic outcomes reinforce competence. At the same time, maintaining engagement with traditional resources helps learners stay grounded in the broader clinical curriculum.

### Limitations

Several limitations merit discussion.

1. Learning approach was defined by self-report, which is vulnerable to recall and social desirability bias. Cross-verification with AI use frequency helped but relied on the same mechanism.
2. The non-randomized design limits causal inference. We cannot determine whether AI-assisted learning itself improved accuracy, or whether pre-existing intern characteristics drove both AI adoption and better performance. Prospective randomized trials would strengthen causal claims.
3. This was a single-center study, and the patient population, case mix, and available AI tools may differ from other institutions. Multi-site replication is needed. Although all interns completed mandatory AI safety training, we did not formally assess their AI literacy. Future studies should measure AI literacy as a potential effect modifier.
4. The intern assessment examinations included both authentic patient encounters and standardized simulated patient scenarios. Although simulated cases were designed by experienced faculty to closely replicate real acute abdomen presentations, they may differ from genuine patient encounters in terms of clinical complexity. If simulated cases systematically differed in difficulty or fidelity from real cases, this could introduce case-type-related bias into the accuracy estimates.
5. Regarding generalizability, our findings apply most directly to emergency medicine training programs in tertiary care hospitals where acute abdomen cases are common and LLM tools are accessible. Extrapolation to other settings may be limited by several factors: the patient population at a single high-volume center may differ from community hospitals or rural settings; the specific AI tools available may have different capabilities than other LLMs; and the Chinese medical education context may differ from training systems in other countries. Multi-site replication studies across diverse healthcare systems are needed.

## Conclusion

AI-assisted and blended learning were both associated with better diagnostic accuracy than textbook-based learning for acute abdominal conditions, with blended strategies showing the strongest effect—particularly for severe and atypical cases. These findings suggest that AI tools are most beneficial when integrated into emergency training as a complement to, rather than a replacement for, established educational resources.

## Data Availability

The raw data supporting the conclusions of this article will be made available by the authors, without undue reservation.
