# Supplementary material for: AI-assisted vs. textbook-based vs. blended learning for acute abdomen diagnosis: a retrospective cohort study of emergency interns
Source: Front Public Health. 2026 Sep 8;14:1914097. doi: 10.3389/fpubh.2026.1914097 (PMC13593857; doi:10.3389/fpubh.2026.1914097)
Supplement: Supplementary file 1 [file Table_1.DOCX]

**Supplementary Table S1.** Case Severity and Typical Presentation Classification Criteria for Each Acute Abdominal Condition

| **Condition** | **Typical Presentation Criteria (≥ 3 of 5 features required)** | **Severity Classification** |
| --- | --- | --- |
| Acute Appendicitis | 1. Migratory right lower quadrant pain 2. Rebound tenderness 3. Fever (> 38°C) 4. Leukocytosis (> 10000/μL) 5. Nausea/vomiting | Mild: Localized RLQ pain, stable vital signs, no systemic inflammatory response Moderate: Diffuse or worsening pain, low-grade fever or mild tachycardia Severe: Hemodynamic instability, peritoneal signs, or organ dysfunction |
| Acute Cholecystitis | 1. Right upper quadrant pain 2. Murphy's sign positive 3. Fever (> 38°C) 4. Leukocytosis (> 10000/μL) 5. Positive ultrasound findings (gallbladder wall thickening, pericholecystic fluid, or gallstones with sonographic Murphy's sign) | Mild: Localized RUQ pain, stable vital signs, no systemic inflammatory response Moderate: Diffuse or worsening pain, low-grade fever or mild tachycardia Severe: Hemodynamic instability, peritoneal signs, or organ dysfunction |
| Intestinal Obstruction | 1. Colicky abdominal pain 2. Abdominal distension 3. Vomiting 4. Obstipation (failure to pass gas or stool) 5. Imaging confirmation (dilated bowel loops with air-fluid levels on abdominal X-ray or CT) | Mild: Localized pain, stable vital signs, no systemic inflammatory response Moderate: Diffuse or worsening pain, low-grade fever or mild tachycardia Severe: Hemodynamic instability, peritoneal signs, or organ dysfunction |
| Urolithiasis | 1. Acute flank pain radiating to groin 2. Hematuria (microscopic or gross) 3. Nausea/vomiting 4. Restlessness (inability to find a comfortable position) 5. Imaging confirmation (stone visualized on CT, ultrasound, or X-ray) | Mild: Localized pain, stable vital signs, no systemic inflammatory response Moderate: Diffuse or worsening pain, low-grade fever or mild tachycardia Severe: Hemodynamic instability, peritoneal signs, or organ dysfunction |
| Ectopic Pregnancy | 1. Amenorrhea or irregular menstruation 2. Lower abdominal pain 3. Vaginal bleeding 4. Positive serum β-hCG 5. Transvaginal ultrasound findings (adnexal mass, empty uterus, or free pelvic fluid) | Mild: Localized pain, stable vital signs, no systemic inflammatory response Moderate: Diffuse or worsening pain, low-grade fever or mild tachycardia Severe: Hemodynamic instability, peritoneal signs, or organ dysfunction |
| Gastric Perforation | 1. Sudden onset severe epigastric pain 2. Board-like abdominal rigidity 3. Free intraperitoneal air on upright chest/abdominal X-ray 4. History of peptic ulcer disease or NSAID use 5. Systemic signs (tachycardia, fever) | Mild: Localized pain, stable vital signs, no systemic inflammatory response Moderate: Diffuse or worsening pain, low-grade fever or mild tachycardia Severe: Hemodynamic instability, peritoneal signs, or organ dysfunction |
| Acute Pancreatitis | 1. Epigastric pain radiating to the back 2. Nausea and vomiting 3. Serum lipase or amylase > 3× upper limit of normal 4. Tenderness in the epigastrium 5. CT findings of pancreatic inflammation (when performed) | Mild: Localized pain, stable vital signs, no systemic inflammatory response Moderate: Diffuse or worsening pain, low-grade fever or mild tachycardia Severe: Hemodynamic instability, peritoneal signs, or organ dysfunction |
| Acute Diverticulitis | 1. Left lower quadrant pain 2. Fever (> 38°C) 3. Leukocytosis (> 10,000/μL) 4. CT findings of colonic diverticula with surrounding fat stranding 5. Change in bowel habits | Mild: Localized pain, stable vital signs, no systemic inflammatory response Moderate: Diffuse or worsening pain, low-grade fever or mild tachycardia Severe: Hemodynamic instability, peritoneal signs, or organ dysfunction |

Case severity was defined based on a standardized clinical scoring framework adapted from the 2013 WSES guidelines for management of intra-abdominal infections. A case was classified as 'typical presentation' when ≥ 3 of the 5 listed classic clinical features were present. Severity and typicality were independently assessed by two board-certified emergency medicine attending physicians blinded to intern learning group allocation. Inter-rater agreement: Cohen's κ = 0.82 (95% CI: 0.76–0.88) for severity; κ = 0.78 (95% CI: 0.71–0.85) for typicality.
