# Supplementary material for: AI-assisted vs. textbook-based vs. blended learning for acute abdomen diagnosis: a retrospective cohort study of emergency interns
Source: Front Public Health. 2026 Sep 8;14:1914097. doi: 10.3389/fpubh.2026.1914097 (PMC13593857; doi:10.3389/fpubh.2026.1914097)
Supplement: Supplementary file 2 [file Table_2.DOCX]

| **Condition** | **Textbook (*n* = 27)** | **AI-Assisted (*n* = 21)** | **Blended (*n* = 24)** | **Total** |
| --- | --- | --- | --- | --- |
| Appendicitis | 32 (11.9%) | 25 (11.9%) | 26 (10.8%) | 83 (11.5%) |
| Cholecystitis | 30 (11.1%) | 27 (12.9%) | 28 (11.7%) | 85 (11.8%) |
| Intestinal obstruction | 29 (10.7%) | 30 (14.3%) | 30 (12.5%) | 89 (12.4%) |
| Urolithiasis | 40 (14.8%) | 32 (15.2%) | 32 (13.3%) | 104 (14.4%) |
| Ectopic pregnancy | 30 (11.1%) | 28 (13.3%) | 30 (12.5%) | 88 (12.2%) |
| Gastric perforation | 25 (9.3%) | 28 (13.3%) | 29 (12.1%) | 82 (11.4%) |
| Pancreatitis | 27 (10.0%) | 26 (12.4%) | 29 (12.1%) | 82 (11.4%) |
| Diverticulitis | 37 (13.7%) | 36 (17.1%) | 34 (14.2%) | 107 (14.9%) |
| *Not applicable* | 20 (7.4%) | 18 (8.6%) | 32 (13.3%) | 70* |
| **Total decisions** | 270 | 210 | 240 | 720 |

**Supplementary Table S2. Case-Mix Distribution by Learning Approach.**

Values shown as *n* (%). Percentages within each column represent the proportion of each condition among total decisions for that group. Chi-square test for distribution across groups: χ² = 3.21, *df* = 14, *p* = 0.995. *Additional decisions not classifiable into the eight predefined conditions were excluded from case-mix analysis.
