# Supplementary material for: AI-assisted vs. textbook-based vs. blended learning for acute abdomen diagnosis: a retrospective cohort study of emergency interns
Source: Front Public Health. 2026 Sep 8;14:1914097. doi: 10.3389/fpubh.2026.1914097 (PMC13593857; doi:10.3389/fpubh.2026.1914097)
Supplement: Supplementary file 3 [file Table_3.DOCX]

# STROBE Statement Checklist

Strengthening the Reporting of Observational Studies in Epidemiology (STROBE)

**AI-Assisted vs. Textbook-Based vs. Blended Learning for Acute Abdomen Diagnosis:
A Retrospective Cohort Study of Emergency Interns**

| **Item** | **Item & Recommendation** | **STROBE Description** | **Location in Manuscript** |
| --- | --- | --- | --- |
| **Title and abstract** |  |  |  |
| **1** | Title and abstract | (a) Indicate the study's design with a word or phrase in the title (b) Provide a summary of key information in the abstract | Title: Page 1, line 1 — "A Retrospective Cohort Study of Emergency Interns"  Abstract: Page 2 — Structured abstract with Background, Objective, Methods, Results, and Conclusions |
|  |  | (c) Ensure the abstract reflects the content of the main text | Abstract accurately reflects findings: Page 2 |
| **Introduction** |  |  |  |
| **2** | Background | Provide the scientific background and rationale, including current knowledge on the topic and gaps that the study addresses | Introduction: Pages 3–5 Paragraphs describe: (1) acute abdomen burden (ref 1), (2) traditional medical education (ref 2), (3) LLM emergence in medicine (refs 3–5), (4) AI in emergency surgery (refs 6–7), (5) blended learning evidence (ref 8), (6) cognitive error reduction hypothesis (refs 9–16) |
| **3** | Objectives | State the specific objectives of the study or hypotheses | Page 5, paragraph beginning "We conducted this study to compare diagnostic accuracy across three learning approaches..." Hypothesis stated: "We hypothesized that AI-assisted and blended learning would be associated with higher diagnostic accuracy than textbook-based learning" |
| **Methods** |  |  |  |
| **4** | Study design | Indicate the study design with key elements stated early in the Methods section | Study Design and Setting: Page 6 "We conducted a retrospective observational cohort study at the tertiary care hospital emergency department" |
| **5** | Setting | Describe the setting, locations, and relevant dates including periods of recruitment, exposure, follow-up, and data collection | Page 6: "June 2025 through June 2026" at a "tertiary care hospital emergency department" "The emergency department manages approximately 70,000 visits a year" Ethics approval: MRCTA,ECFAH of FMU(2026)704 (Page 6) |
| **6** | Participants | (a) Eligibility criteria for participants and sources (b) Methods of setting selection | Page 7: "All interns rotating through the emergency department during the study period were consecutively enrolled" Total: 72 interns, each contributing 8–12 clinical decisions (720 total) Three groups defined: textbook-based, AI-assisted, blended (Page 7) Group classification cross-verified with self-reported AI use frequency |
| **7** | Variables | (a) Clearly define all outcomes, exposures, predictors, potential confounders, and effect modifiers (b) Data sources for each variable | Primary outcome: diagnostic accuracy (binary), defined as concordance with final verified diagnosis — Page 8 Secondary outcomes: diagnosis time, missed diagnosis, misdiagnosis — Page 8 Exposure: learning approach (textbook/AI/blended) — Page 9 Covariates: age, gender, education duration, rotation experience, baseline score, severity, typicality, imaging availability — Page 9 Case severity and typicality definitions — Pages 9–10 Condition difficulty ratings — Page 10 |
| **8** | Data sources/measurement | (a) For each variable, give the source of data and details of methods of assessment (b) Describe comparability of assessment methods if there's more than one group | Data Sources and Measurement: Pages 8–9 Diagnostic decision data from departmental database — Page 8 Faculty supervision documentation process — Page 8 Mixed case types (~65% real patients, ~35% simulated) — Page 8 Intern demographics from medical education database and pre-rotation survey — Page 9 Same assessment instruments used for all groups; supervised by faculty — Pages 8–9 |
| **9** | Bias | Describe any efforts to address potential sources of bias | Several strategies described: • Cross-verification of learning approach with AI use frequency — Page 7 • Propensity score matching at intern level to reduce confounding — Page 11 • Severity and typicality assessed independently by two board-certified attendings (blinded to group allocation) — Page 10 • Inter-rater reliability reported: κ = 0.82 (severity), κ = 0.78 (typicality) — Page 10 • Case-mix balance checked (Supplementary Table S2) — Page 12 • E-values calculated for unmeasured confounding — Page 12 |
| **10** | Study size | Explain how the study size was determined | Page 11, Statistical Analysis section: Consecutive enrollment design "A total of 72 emergency interns were included in the analysis, contributing 720 clinical decisions" All available interns during study period were enrolled; no formal sample size calculation described Post hoc power analysis mentioned: 89% for blended vs textbook, 74% for AI vs textbook — Page 12 |
| **11** | Quantitative variables | Explain how quantitative variables were handled in the analyses. If applicable, describe which groupings were chosen and why | Page 9: Continuous variables summarized as mean ± SD (age, education duration, baseline score) Baseline score categorized into tertiles for subgroup analysis — Page 12 Diagnosis time analyzed as continuous (Kruskal-Wallis test) — Page 13 Propensity scores ranged 0.11–0.74 (AI vs Text) and 0.09–0.78 (Blend vs Text) — Pages 11–12 |
| **12** | Statistical methods | (a) Describe all statistical methods used to control for confounding (b) Describe any methods used to examine subgroups and interactions (c) Explain how missing data were addressed (d) If applicable, explain how loss to follow-up was addressed (e) Describe any sensitivity analyses | Statistical Analysis: Pages 11–12 (a) PSM at intern level with pair-stratified GEE; multivariable logistic regression adjusting for all covariates — Pages 11 (b) Predefined subgroup analyses by condition, severity, typicality, imaging, baseline tertile, AI frequency; FDR correction applied — Pages 12, 32–33 (c) No missing data; complete case analysis — Page 9: "No data were missing for any variable" (d) Not applicable (retrospective study with complete records) (e) E-value analysis, multivariable adjustment, GEE with exchangeable correlation, PSM — Pages 11–12, 27–28 |
| **Results** |  |  |  |
| **13** | Participants | (a) Report numbers of individuals at each stage of the study (e.g., numbers potentially eligible, examined for eligibility, confirmed eligible, included in the study, completing follow-up, and analyzed) (b) Give reasons for non-participation at each stage | Study Population: Page 13 "A total of 72 emergency interns were included in the analysis, contributing 720 clinical decisions (mean 10 decisions per intern)" All 72 enrolled interns completed the rotation and contributed data Figure 1: Study Flow Diagram — shows participant selection, group assignment, PSM, and analysis sets No exclusions or loss to follow-up reported |
| **14** | Descriptive data | (a) Give characteristics of study participants (e.g., demographic, clinical, social) (b) Give information on exposures and potential confounders (c) Indicate number of participants with missing data for each variable | Table 1: Baseline Characteristics by Learning Approach — Page 14 Includes: age, gender, education duration, rotation months, baseline score, prior rotations, learning style, AI use frequency Pre-matching differences noted: blended learners had higher baseline scores (p = 0.002) and more rotation months (p = 0.004) — Page 14 Tables 3–4: Post-matching characteristics showing balance — Pages 15–16 Missing data: None — Page 9: "No data were missing for any variable included in the analysis" |
| **15** | Outcome data | (a) Report numbers of events or summary measures for each group (b) If applicable, report the precision of the estimates (e.g., 95% confidence intervals) | Table 2: Diagnostic Accuracy by Learning Approach — Page 15 Textbook: 73.0% (197/270), AI: 82.4% (182/220), Blended: 221/230 (87.1%) Overall χ² = 16.81, df = 2, p < 0.001 95% CIs reported for all group proportions Secondary outcomes reported on Page 33: diagnosis time, missed diagnosis, misdiagnosis rates |
| **16** | Main results | (a) Give unadjusted estimates and, if applicable, confounder-adjusted estimates and their precision (e.g., 95% confidence intervals) (b) Categorize continuous variables or create subgroups if appropriate | Unadjusted: Page 16 — crude ORs reported Adjusted (PSM-GEE): Pages 16–17 — pair-stratified GEE with 95% CIs Multivariable: Page 27 — adjusted ORs with 95% CIs (Table 5) Key results: AI vs Text OR = 1.38 (95% CI: 0.67–2.83), p = 0.379; Blend vs Text OR = 2.84 (95% CI: 1.27–6.36), p = 0.011 E-values: AI = 2.75, Blend = 5.88 — Page 28 |
| **17** | Other analyses | Report other analyses done — e.g., analyses of subgroups and interactions, and sensitivity analyses | Sensitivity analyses: Table 5, Page 27 — Multivariable logistic regression, GEE with exchangeable correlation Subgroup analyses: Table 6, Pages 32–33 — by condition, severity, typicality, imaging, baseline tertile, AI frequency Figure 2: Forest Plot of Subgroup Analyses — Page 41 FDR-corrected q-values for all subgroup comparisons Case-mix distribution: Supplementary Table S2 — Page 33 Secondary outcomes: Page 33 — diagnosis time, missed/misdiagnosis rates |
| **Discussion** |  |  |  |
| **18** | Key results | Summarize key results with reference to study objectives | Discussion: Pages 34–35 Opening paragraph summarizes: both AI-assisted and blended learning associated with higher accuracy; blended strongest Clinical significance: "14-point improvement means among every seven patients... one additional correct diagnosis" — Page 35 |
| **19** | Limitations | Discuss limitations of the study, taking into account sources of potential bias or inaccuracy. Give the direction and magnitude of any potential bias | Limitations section: Pages 38–40 • Self-report exposure classification (recall/social desirability bias) — Page 38 • Residual confounding (E-value 5.88 for blend) — Page 38 • Non-randomized design limits causal inference — Page 39 • Modest sample (72 interns, 11/13 matched pairs) — Page 39 • Single-center — Page 39 • Mixed real/simulated cases — Page 39 • Generalizability limited to tertiary care EDs — Page 40 Direction and magnitude of bias discussed via E-values |
| **20** | Interpretation | Give a cautious overall interpretation of results in light of study objectives, limitations, multiplicity of analyses, results from similar studies, and other relevant evidence | Discussion: Pages 34–38 Comparison with literature: Pages 35–36 — discusses consistency with prior studies (refs 3–8, 13–16) Implications for medical education: Pages 36–37 Implications for learning theory: Pages 37–38 — cognitive load theory, self-determination theory Cautionary notes: "None of these findings argue for uncritical reliance on AI" — Page 37 Addresses multiplicity: FDR correction for subgroup analyses — Page 33 |
| **21** | Generalizability | Discuss the generalizability of the study results | Page 40: "our findings apply most directly to emergency medicine training programs in tertiary care hospitals where acute abdomen cases are common and LLM tools are accessible" Notes that extrapolation to other specialties, levels of training, and institutional contexts requires further study Suggests multi-site replication needed |
| **Other information** |  |  |  |
| **22** | Other information | (a) Give the source of funding and the role of funders for the present study and, if applicable, for the original study on which the present article is based (b) Give or declare any conflict of interest for each author | Funding: Page 41 — "This study was supported by the Fujian Medical University Startup Fund. The funder had no role in the design, conduct, or reporting of this study."  Conflict of Interest: Page 41 — "The authors declare that the research was conducted in the absence of any commercial or financial relationships that could be construed as a potential conflict of interest."  Ethics Approval: Page 41 — IRB approval MRCTA,ECFAH of FMU(2026)704  Data Availability: Page 41 — available from corresponding author on reasonable request  STROBE Compliance: Page 41 — "The study was conducted and reported in accordance with the STROBE statement" |
